# Dual Targeting by Inhibition of Phosphoinositide-3-Kinase and Mammalian Target of Rapamycin Attenuates the Neuroinflammatory Responses in Murine Hippocampal Cells and Seizures in C57BL/6 Mice

**DOI:** 10.3389/fimmu.2021.739452

**Published:** 2021-11-23

**Authors:** Preeti Vyas, Rajkumar Tulsawani, Divya Vohora

**Affiliations:** ^1^ Department of Pharmacology, School of Pharmaceutical Education and Research, Jamia Hamdard, New Delhi, India; ^2^ Defense Institute of Physiology & Allied Science, Defense Research and Development Organization, New Delhi, India

**Keywords:** phosphoinositide-3-kinase (PI3K), mammalian target of rapamycin (mTOR), neuronal inflammation, lipopolysaccharide, pilocarpine, cytokines, PI3K/Akt/p-p70S6 pathway, kinase inhibition

## Abstract

Emerging evidence suggests the association of seizures and inflammation; however, underlying cell signaling mechanisms are still not fully understood. Overactivation of phosphoinositide-3-kinases is associated with both neuroinflammation and seizures. Herein, we speculate the PI3K/Akt/mTOR pathway as a promising therapeutic target for neuroinflammation-mediated seizures and associated neurodegeneration. Firstly, we cultured HT22 cells for detection of the downstream cell signaling events activated in a lipopolysaccharide (LPS)-primed pilocarpine (PILO) model. We then evaluated the effects of 7-day treatment of buparlisib (PI3K inhibitor, 25 mg/kg p.o.), dactolisib (PI3K/mTOR inhibitor, 25 mg/kg p.o.), and rapamycin (mTORC1 inhibitor, 10 mg/kg p.o.) in an LPS-primed PILO model of seizures in C57BL/6 mice. LPS priming resulted in enhanced seizure severity and reduced latency. Buparlisib and dactolisib, but not rapamycin, prolonged latency to seizures and reduced neuronal loss, while all drugs attenuated seizure severity. Buparlisib and dactolisib further reduced cellular redox, mitochondrial membrane potential, cleaved caspase-3 and p53, nuclear integrity, and attenuated NF-*κ*B, IL-1β, IL-6, TNF-α, and TGF-β1 and TGF-β2 signaling both *in vitro* and *in vivo* post-PILO and LPS+PILO inductions; however, rapamycin mitigated the same only in the PILO model. Both drugs protected against neuronal cell death demonstrating the contribution of this pathway in the seizure-induced neuronal pyknosis; however, rapamycin showed resistance in a combination model. Furthermore, LPS and PILO exposure enhanced pAkt/Akt and phospho-p70S6/total-p70S6 kinase activity, while buparlisib and dactolisib, but not rapamycin, could reduce it in a combination model. Partial rapamycin resistance was observed possibly due to the reactivation of the pathway by a functionally different complex of mTOR, i.e., mTORC2. Our study substantiated the plausible involvement of PI3K-mediated apoptotic and inflammatory pathways in LPS-primed PILO-induced seizures and provides evidence that its modulation constitutes an anti-inflammatory mechanism by which seizure inhibitory effects are observed. We showed dual inhibition by dactolisib as a promising approach. Targeting this pathway at two nodes at a time may provide new avenues for antiseizure therapies.

## 1 Background

The PI3K/Akt/mTOR signaling pathway controls various cell-signaling events involved in neuronal proliferation, growth, and survival (1–3). While the role of mammalian target of rapamycin (mTOR) in epileptic seizures is well established (4–6), the role of phosphoinositide-3-kinases (PI3K), upstream of mTOR, in seizures is only recently getting evidenced. In the past few years, the direct effects of PI3K inhibition have been demonstrated in an organotypic hippocampal culture model (7), seizures associated with brain overgrowth disorders (8), and electroconvulsive seizures (9). Contradictory reports also suggest a decrease in hippocampal phosphoinositoide-3,4,5-triphosphate (PIP3) and protein kinase B (PKB or Akt) phosphorylation (10) upon kainic acid administration, a chemical convulsant. A recent study, however, has shown no antiseizure effects of PI3K/mTORC1/2 inhibition on the kainate model (11). On the other hand, there are studies that delineate the role of mTOR, a downstream mediator of PI3K, in epileptic seizures. The mTOR inhibitors protect, in a mouse model of the *TSC* complex (12), kainate as well as pilocarpine (PILO, another chemical convulsant) models (13) and also suppress mossy fiber sprouting (14). These contrasting evidences demonstrate that the current picture of the involvement of this PI3K/Akt/mTOR pathway and related inhibitors in seizures is not clear. It is still a major concern how this PI3K signaling should be targeted or which downstream target should be modulated to complement the PI3K inhibition in seizures.

Various preclinical as well as clinical observations (15, 16) associate the inflammatory pathways with seizures. Alongside, recent literature has highlighted a shared role of PI3Ks and mTOR in the pathophysiology of neuronal inflammation as well as seizures (2, 17–20). It has been demonstrated that the peripheral inflammatory reactions lower the seizure threshold in a time-dependent manner in rodents. PILO administration, known to evoke prolonged seizures, activates the neuroinflammatory pathways in the brain (21). The peripheral administration of lipopolysaccharide (LPS), which is a bacterial endotoxin, is also known to induce neuroinflammatory pathways (22, 23) (24, 25). A previous study in our laboratory has shown that LPS priming prior to PILO results in overactivation of inflammatory as well as the seizure-related pathways in the brain. The peripheral administration of LPS 2 h before the PILO administration in mice led to the complete loss of the efficacy of sodium valproate (SVP) and carbamazepine (CBZ), and a partial loss in the efficacy of levetiracetam (LEV) (26). Our work mandated the further exploration of other cell signaling pathways, particularly PI3K/Akt signaling and their inhibitors, in neuronal inflammation-mediated seizures.

Buparlisib (BUP), the most clinically advanced pan-PI3K-inhibitor for cancer, has shown efficacy in the pentylenetetrazole (PTZ) model of seizures associated with brain overgrowth disorders (8); however, its antiseizure potential has never been studied in other models. Also, considering its high oral bioavailability and lipophilicity, we have used the same to pharmacologically inhibit PI3K in our study. We have also used dactolisib (DACT), a dual PI3K/mTORC1/2 inhibitor, in our work to further see if inhibiting the PI3K pathway at two nodes shows any potentiation in the antiseizure potential. DACT has shown the anti-epileptogenic and neuroprotective potential in an organotypic hippocampal culture model (7); however, its antiseizure potential has never been studied using *in vivo* models.

In the present work, we have combined LPS and PILO as inducers of neuronal inflammation and seizures to mimic the actual inflammation-mediated seizure pathology in the brain as previously reported (26), and we have studied the involvement of PI3K/Akt signaling in the same. Herein, the *in vitro* studies were also carried out to study the underlying inflammatory cell signaling pathways using mouse hippocampal HT-22 neuronal cells. Furthermore, we evaluated the protective role of the PI3K inhibitor (BUP), the mTOR inhibitor (RAPA), and their dual inhibition (DACT) on neuronal inflammation-mediated seizures in PILO as well as LPS-primed PILO models *in vivo*. Our findings indicate that PI3K/Akt signaling is an important pathway involved in neuronal inflammation and neurodegenerative changes associated with seizures, and its inhibition supports antiseizure and neuroprotective effects.

## 2 Materials and Methods

### 2.1 Drugs and Reagents

Solutions of pilocarpine (PILO), lipopolysaccharide (LPS; from *Escherichia coli*), buparlisib (BUP; BKM-120; Pan-PI3K inhibitor), dactolisib (DACT; BEZ-235, PI3K/mTOR dual inhibitor), and rapamycin (RAPA; Sirolimus; mTOR inhibitor) were prepared in pyrogen-free normal saline for all the experiments. Other research-grade chemicals used in this study were purchased from Sigma.

### 2.2 The *In Vitro* Studies

#### 2.2.1 Cell Culture and Treatments

HT22 cells (subclone of HT4 mouse hippocampal cell line, received as a kind gift of Dr. Dave Schubert, Salk Institute, San Diego, CA, USA) were cultured and grown into DMEM-high glucose media supplemented with 10% heat-inactivated fetal bovine serum or FBS and 1% antibiotic antimycotic solution. The cells were incubated at 37°C with 5% CO_2_ using a New Brunswick Galaxy 48R CO_2_ incubator. The cells were exposed to the test compounds, followed by stimulation with the optimized concentration of LPS after 30 min and/or PILO after 1 h subsequent to drug treatment (27).

#### 2.2.2 Sample Preparation for Cell-Based Studies

The cell supernatants were collected after 24 h of treatments, and the samples were prepared using 0.5 M phosphate buffer or the RIPA buffer (150 mM sodium chloride, 1.0% NP-40, 0.5% sodium deoxycholate, 0.1% SDS; sodium dodecyl sulfate, 50 mM Tris, pH 8.0) supplemented with protease inhibitor cocktail and PMSF.

#### 2.2.3 Detection of Neuronal Cell Death

After 24 h of drug treatment, the cells were gently washed using I X phosphate buffer saline (PBS; 10 mM, 2.7 mM potassium chloride, 137 mM sodium chloride, and 1.76 mM potassium phosphate, pH 7.4 ± 0.2), and subsequently, 50 μl of 1 mg/ml of methyl thiazole tetrazolium (MTT) solution was added. After 2–4 h of incubation, the formazan crystals were solubilized using 100 μl of DMSO and incubated in shaking conditions for 5 min to dissolve the formazan crystals. Subsequently, the absorbance was measured at 570 nm with 630 nm as a reference filter. Absorbance measured in untreated cells was taken as 100% survival (28).

#### 2.2.4 Determination of the Mitochondrial Membrane Potential (MMP; ΔΨm)

The HT22 cells were seeded at the cell density of 1x10^3^ cells/well in a 96-well plate. After 24 h, they were treated with the optimized concentrations of drugs and inducers. Subsequent to 24 h of treatments, 10 µl of Rhodamine 123 dye (10 µg/ml) was added to the cells and incubated for 30 min. Later, the cells were washed thrice with 1X PBS gently and then lysed with 1% NP-40 buffer. Fluorescence was measured using the plate reader at Ex/Em of 485/530 nm (29).

#### 2.2.5 Quantification of Active Caspase-3 Levels

For the quantification of caspase-3 levels, the adherent HT22 cells were grown in the high glucose-DMEM media (containing 10% heat-inactivated FBS and 1% antibiotic) and were seeded at the cell density of 1x10^6^ cells/plate in 6-well plates for 24 h following the treatments. Caspase-3 levels were measured using a CaspGLOW™ fluorescein active caspase-3 staining kit. In this assay, a caspase-3 inhibitor, Z-VAD-FMK (1 µl/ml), was also added as a negative control to the induced cells to inhibit the caspase-3 activation. After 24 h of treatment, the cells were trypsinized and resuspended in 1X PBS and labeled with FITC. The cells were incubated for 30 min at 37°C with 5% CO_2_ and washed three times. For fluorescence analysis, the cells were then transferred to a black microtiter plate, and fluorescence intensity was measured at Ex/Em of 485/535 nm. For control, the wells containing unlabeled cells were used.

#### 2.2.6 Measurement of Cellular Lactate Dehydrogenase (LDH) Leakage

For LDH estimation, HT22 cells were plated in 96-well plates at a cell density of 8 X 10^3^ cells/well in complete DMEM high glucose media (containing 10% heat-inactivated FBS and 1% antibiotic). After 24 h of drug treatment, cell supernatants were collected for the estimation of extracellular LDH levels. Next, the adhered cells were lysed using 1% NP-40 cell lysis buffer (50 mM Tris-Cl; pH=8.0, 150 mM NaCl, 1% NP-40) (28). After the addition of buffer to the wells, the plates were incubated at 4°C for 20 min with occasional pipetting. The prepared lysates were collected in centrifuge tubes and centrifuged at 8,000 rpm for 20 min. Supernatants were collected for the estimation of intracellular LDH. The LDH leakage was calculated as follows:

LDH leakage = (Extracellular LDH/Total LDH) X 100

where Total LDH=Intracellular LDH + Extracellular LDH.

#### 2.2.7 Determination of Nuclear Integrity

HT22 cells were seeded at a cell density of 1x10^3^ cells/well. At a similar time point as above, 10 µl of propidium iodide (30 µg/ml) was added to the cells and incubated for 30 min. Later, the cells were washed thrice using 1X PBS and lysed with 1% NP-40 buffer. Fluorescence was measured using the plate reader at Ex/Em of 535/614 nm. Images were captured using the fluorescence microscope.

#### 2.2.8 Measurement of Intercellular Reactive Oxygen Species (ROS) Burden and Markers of Oxidative Stress

For ROS estimation, the adherent HT22 cells were grown and treated as discussed in study design. At the end of the incubation period of 24 h, the media was removed and 100 μl/well of 1X PBS was added to gently wash the cells. The buffer was removed and 100 μl of 10 μM of 2′,7′-dichlorofluorescin diacetate (DCFDA) solution was added per well, and the plates were incubated at 37°C with 5% CO_2_ for 30–40 min in the dark. Subsequently, the fluorescence was taken with an excitation wavelength of 485 nm and an emission wavelength of 530 nm using Spectra Max Gemini EM (28). Other biomarkers of oxidative stress, i.e., the levels of malondialdehyde (MDA) (30), nitrite–nitrate, reduced glutathione (GSH), and super-oxide dismutase (SOD), were measured (31) using standard protocols.

#### 2.2.9 Biochemical Estimations

pAkt/Akt, phospho-pS6/Total-pS6 kinase, pERK/ERK, serum, and hippocampal cytokines (mouse IL-1β, TNF-α, IL-6, TGF-β1, TGF-β3) were measured using commercially available ELISA kits.

### 2.3 Neuroinflammation-Mediated Model of Seizures

#### 2.3.1 Animals

Adult C57BL/6 male mice (20–25 g) having free access to a standard pellet diet (Amrut rat and mice feed, Chakan oil mills, Pune, India) and tap water *ad libitum* were used. Post the day of induction, the animals were provided with glucose solution in addition to tap water to replenish the fluid content in the body (32). Male mice were used in the study to limit the influence of modulatory effects of estradiol on seizures.

The sample size was calculated using the G* power software (version 3.1.9.3 for Mac OS) based on a previous study carried out in our laboratory. The type I error (alpha) was 0.05, and the power was 0.95. The allocation ratio (n_1_/n_2_) was 1. The computed effect size was 2.54, and the sample size was calculated to be 6 in each group. Groups of equal sizes were generated using randomization, and behavioral analysis was performed by blinded experimenters in a soundproof room of the Neurobehavioral Pharmacology Laboratory of Jamia Hamdard, New Delhi.

#### 2.3.2 The *In Vivo* Experimental Design

**Figure 1:**
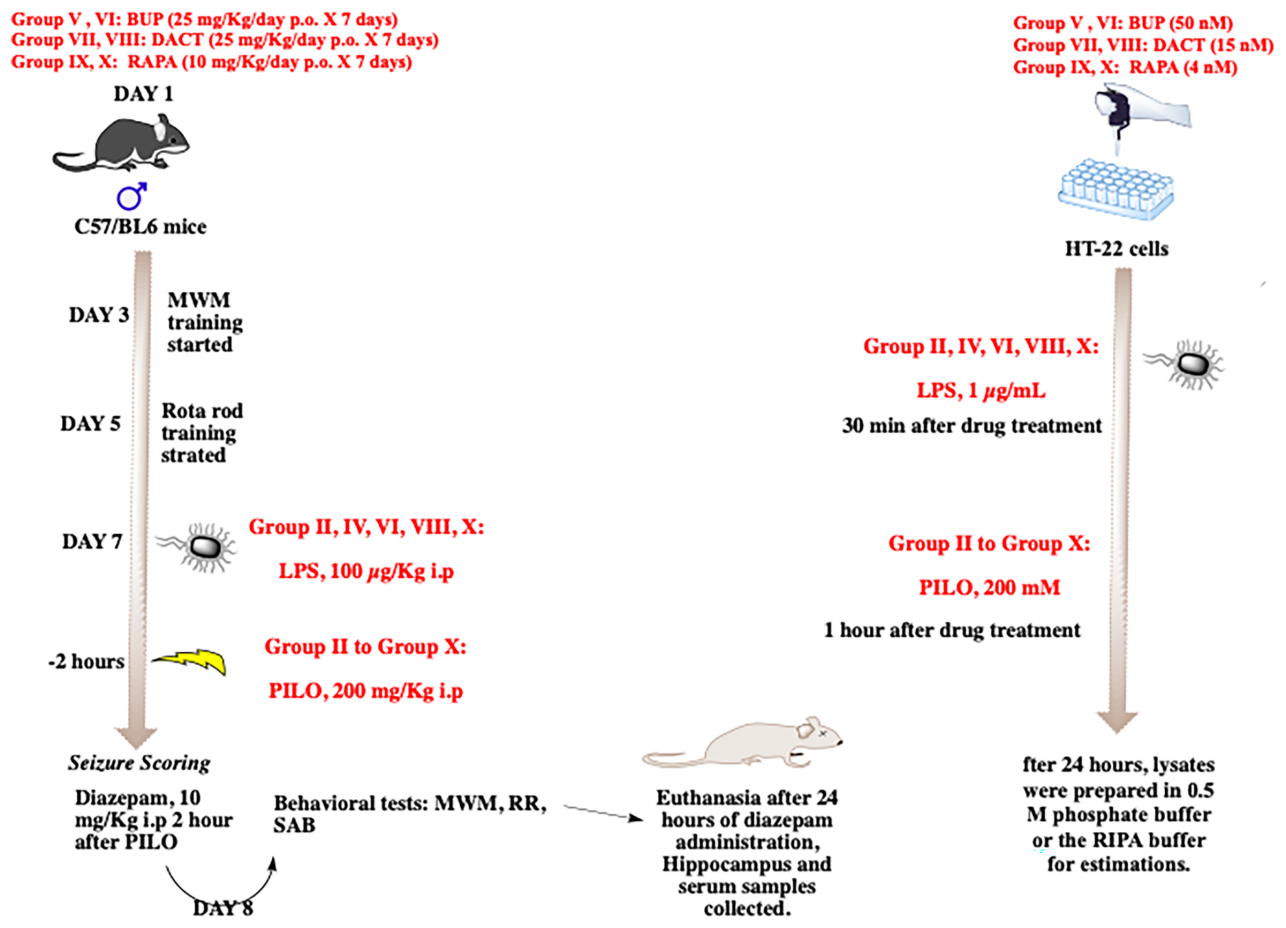
The *in vitro* and *in vivo* experimental design.

The prepared solutions of BUP (25 mg/kg/day p.o.), DACT (25 mg/kg/day p.o.), and RAPA (10 mg/kg p.o.) were administered for 7 days in a volume not exceeding 10 ml/kg. The groups received the drug treatment for 7 days, and the drugs were administered 30 min prior to PILO or LPS+PILO-induced SE on day 7 of the experiment (**Figure 1**). The p.o. or oral administration of the drugs was carried out using a reusable stainless-steel feeding needle for mice. We ensured that the ball tip passed through the length of the esophagus and was never forcibly used above a predetermined length.

### Animal Treatments

Group I: Normal saline (SAL, 10 mg/kg); Group II: LPS 100 μg/kg i.p.; Group III: PILO 200 mg/kg i.p.; Group IV: LPS (100 μg/kg i.p.) + PILO (200 mg/kg i.p.); Group V: RAPA (10 mg/kg p.o.) + PILO (200 mg/kg i.p.); Group VI: LPS (100 μg/kg i.p.) + RAPA (10 mg/kg/p.o.) + PILO (200 mg/kg i.p.); Group VII: BUP (25 mg/kg/day p.o.) + PILO (200 mg/kg i.p.); Group VIII: BUP (25 mg/kg/day p.o.) + LPS (100 μg/kg i.p.) + PILO (200 mg/kg i.p.); Group IX: DACT (25 mg/kg p.o.) + PILO (200 mg/kg i.p.); Group X: DACT (25 mg/kg p.o.) + LPS (100 μg/kg i.p) + PILO (200 mg/kg i.p.). All doses were selected from the previous preclinical and clinical studies carried out in our as well as other laboratories (33–36).

#### 2.3.3 Behavioral Studies

Based on our previous laboratory experiments, a single dose of PILO 200 mg/kg i.p. was selected to induce the *status epilepticus* (*SE*) with low mortality rates. The LPS+PILO group received an i.p. injection of LPS 2 h prior to the PILO challenge. Prior to seizure induction, we trained the animals for the Morris water maze (5 days) and the rota rod test (3 days), and seizures were induced on the seventh day of experiment by intraperitoneal administration of PILO and LPS+PILO (**Figure 1**). Individual animals were observed for 120 min post administration. They were placed individually in cages, and their measurements were recorded separately, which include the latency to seizure onset and severity. The convulsive activities of the animals were scored based on a slight variation of the Racine scale modified by Borges (37–40), which is as follows: Stage 0—normal activity; Stage 1—rigid posture or immobility; Stage 2—circling movement; Stage 3—stiffened, extended, and often arched (Straub’s) tail; Stage 4—partial body clonus, including forelimb or hind limb clonus, head bobbing, and jerking; Stage 4.5—whole body continuous clonic seizures while retaining posture; Stage 5—rearing; Stage 5.5—severe whole body continuous clonic seizures while retaining posture; Stage 6—rearing and falling; and Stage 7—tonic–clonic seizures with loss of posture or jumping. The person scoring the experiments was blinded with the treatments received by the animals. The constant seizures for 120 min including the stages of no less than 3.5 and/or few stages of 4.5 or a single higher stage was considered as the development of *status epilepticus*. After an interval of 120 min, the seizures were terminated in all mice using the intraperitoneal injection of diazepam (10 mg/kg i.p.). The systemic effects of PILO were antagonized by pretreatment with a peripherally acting muscarinic antagonist methyl-scopolamine (1 mg/kg i.p).

After 24 h of the seizure induction, spontaneous alternation behavior (SAB) (41), rota-rod (42) (43), and Morris water maze tests (41) were performed to evaluate the effects on cognitive and motor functions.

#### 2.3.4 Sample Preparation and Analysis

##### 2.3.4.1 Histopathological Analysis

For histopathological analysis, the sample processing using hematoxylin and eosin (H&E) and 0.1% cresyl violet (CV) staining was carried out using the standard procedures utilized in previous studies conducted in our laboratory (33). The sections were visually examined for neuronal loss, pyknosis, and live cells in the hippocampal formation (CA1, CA3, and DG regions). % Pyknosis was estimated by counting the total number of live cells and dead cells with pyknotic nuclei in the entire optical field of each CA1, CA3, and DG regions (magnification 40X) in each group. An observer blinded to the treatment conditions counted picked the sections randomly. The counting in each field was repeated thrice, and the total score for each animal was obtained from the sum of the score of three areas. The neuronal loss was calculated as the percentage of pyknotic cells (number of pyknotic cells/total number of cells ×100) per counting field (33).

##### 2.3.4.2 Protein Estimations Using ELISA

For the *in vivo* studies, the animals were euthanized, and the brain and blood samples were collected. Serum was collected from the blood sample by centrifuging the blood at 10,000 g for 10 min at 4°C. Supernatants were collected and recentrifuged for a second time for 10 min. The pAkt/Akt, phospho-pS6/Total-pS6 kinase, pERK/ERK, Caspase-3, and pNF-κB levels were measured. In all, the equalized protein samples were used on respective precoated ELISA plates and incubated as per the protocols. Furthermore, HRP-conjugated secondary antibodies were added, and further incubation was carried out. TMB substrate was used for the generation of the color. Reaction was terminated using the stop solution supplied with the kit, and pale yellow color was obtained. The plates were read at 450 nm using an ELISA plate reader. Alongside, serum lactate dehydrogenase (LDH) was also measured in the serum samples of B6 mice using a commercial diagnostic kit.

##### 2.3.4.3 Western Immunoblotting

Western blot analysis was carried out for the detection of p53 levels. The total protein in the samples was measured using the Bradford reagent test, and the samples were prepared. About 20 µg of protein samples was resolved on 10–12% SDS-polyacrylamide gel and was later subsequently transferred onto PVDF membranes. The membranes were incubated with the p53 antibody and β-actin followed by HRP conjugated secondary antibodies. Immunoblots were detected with the pierce enhanced chemiluminescence plus substrate using a luminescence detector, and the band intensities were detected using the Image J software.

##### 2.3.4.4 Cytokine and Other Biochemical Estimations

Hippocampal and serum cytokine levels were also measured in the brain and serum using commercially available ELISA kits. Oxidative stress markers like the levels of malondialdehyde (MDA) (30), nitrite–nitrate, reduced glutathione (GSH), and superoxide dismutase (SOD) activities were measured (31).

### 2.4 Statistical Analysis

The experiments conducted were randomized, and the experimenters were blinded with the treatments received by the cells or animals. Data were represented as recommended by the experimental design and analysis and their reporting (44). All the cell-based experiments were conducted five times independently (not as replicates). The statistical and graphical presentations were carried out in Graph Pad Prism 7.0 for mac OS (Graph Pad Software). Data were presented as mean ± SEM of “n” observations, where the statistical evaluation was performed using one-way or two-way analysis of variance (ANOVA) followed by Dunnett’s *post hoc* test (unless otherwise stated). These *post hoc* tests were applied when “F” achieved the necessary level of statistical significance (i.e., p < 0.05). The level of significance was set at p<0.05. Nonparametric Kruskal–Wallis test, wherever used, is followed by Dunn’s multiple comparison. All the data outliers were included in data analysis and presentation, and no data exclusion criteria were applied.

## 3 Results

### 3.1 The *In Vitro* Studies

#### 3.1.1 PI3K, mTOR, and Their Dual Inhibition Decrease the LPS and/or PILO Induced Cell Death and Redox in HT22 Cells

**Figure 2:**
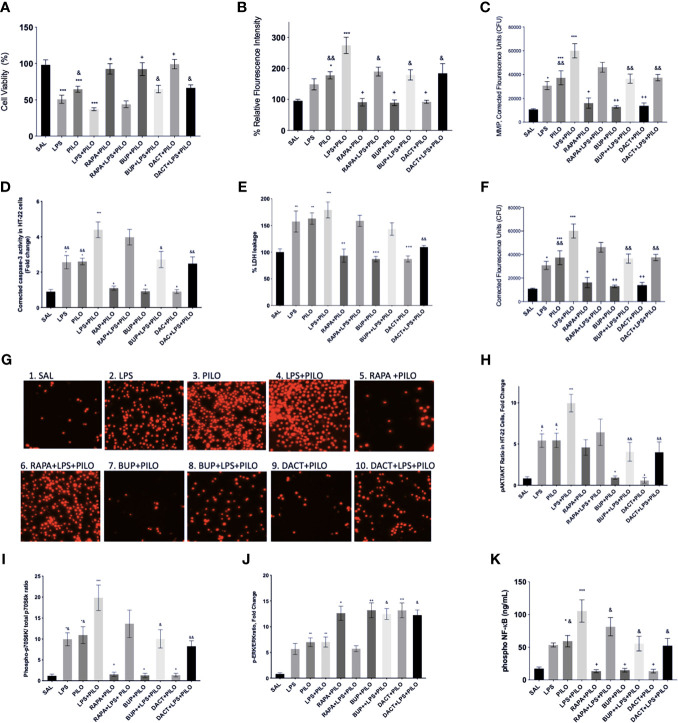
Effects of RAPA, BUP, and DACT on PILO *versus* LPS+PILO-induced HT-22 cells on: **(A)** cell viability, **(B)** ROS levels, **(C)** mitochondrial membrane potential, **(D)** cleaved caspase-3, **(E)** % LDH leakage, **(F)** nuclear integrity, **(G)** fluorescence microscope images of propidium iodide stained HT-22 cells 60X, **(H)** phospho Akt/total Akt ratio in cell lysates, **(I)** phospho-p70S6/Total-p70S6 kinase, **(J)** phospho ERK/total ERK, and **(K)** phospho NF-κB levels (ng/ml). SAL represents the saline-treated wells (n=6). The data are represented as mean ± SEM and analyzed by one-way ANOVA followed by Tukey Kramer multiple comparison tests. The significance is ascertained as ^***^p<0.001 *versus* SAL, ^**^p<0.01 *versus* SAL, ^*^p<0.05 *versus* SAL, ^++^p<0.01 *versus* PILO, ^+^p<0.05 *versus* PILO, ^&&^p<0.01 *versus* LPS+PILO, and ^&^p<0.05 *versus* LPS+PILO.

We evaluated the test drugs for any cytotoxic liability by conducting a cell viability assay using HT22 cells by MTT assay. **Figure 2A** depicts the effects of pharmacological inhibition of PI3K, mTOR, and their dual inhibition in HT22 cells induced with LPS and/or PILO for 24 h. Interestingly, LPS+PILO induced cell death was attenuated upon treatment with BUP (p=0.483), DACT (p=0.0051), and RAPA (p=0.457). However, only DACT (p=0.0285) and BUP (p=0.0458) showed protection in the PILO model (F (9,40)= 15.86). To further understand the contribution of inflammatory mediators in this cell signaling, we tested the cells for the levels of the reactive oxygen species as shown in **Figure 2B**. An increase in the cellular redox was observed on PILO challenge, which was further increased upon pretreatment with LPS. BUP, DACT, and RAPA resulted in a significant decrease in the ROS levels in both PILO (p=0.0210, p=0.0.294, p=0.0256) and LPS+PILO (p=0.0101, p= 0.0178, p=0.0325) models (F (9,40)= 12.8).

#### 3.1.2 PI3K and PI3K/mTOR But Not mTOR Alone Inhibition Attenuates Mitochondrial Membrane Potential (MMP) in LPS+PILO Induced HT-22 Cells

We observed a significant increase in MMP in LPS, PILO, and LPS+PILO treated cells. BUP and DACT reversed the changes in both PILO (p=0.0021, p=0.0034) and LPS+PILO (p=0.0038, p=0.0058) treated cells; however, RAPA could reverse the same only in PILO (p=0.0113) treated cells [F (9,40)= 18.33] (**Figure 2C**).

#### 3.1.3 PI3K and PI3K/mTOR Inhibitors Attenuate Apoptotic and Necrotic Pathways in PILO and LPS+PILO Induced HT-22 Induced Cells

Herein, we observed the increase in caspase-3 in LPS (2.56±0.37 times), PILO (2.6 ±0.18-fold), as well as LPS+PILO (4.40±0.445-fold) treated cells as compared to SAL-treated groups [F (9,40)= 16.94] (**Figure 2D**). The commercial caspase-3 inhibitor decreased the levels to baseline when compared with SAL in both PILO as well as LPS+PILO treated groups confirming the apoptotic cell death (data not shown). BUP (0.913±0.129 fold, 0.0148), DACT (0.900 ±0.12 fold, p=0.0137), and RAPA (1.093±0.109 fold, p=0.0.0430) inhibited the levels of active caspase-3 in the PILO model. However, BUP (2.719±0.459 fold, p=0.0154) and DACT (2.489±0.376 fold, p=0.0035), but not RAPA (3.98±0.438 fold, 0.9935), showed significant attenuation in the LPS+PILO model (**Figure 2D**).

It was also interesting to observe the increase in the LDH leakage in LPS (1.5 ± 0.19 fold), PILO (1.62 ± 0.10 fold), as well as LPS+PILO (1.79±0.14 fold) treated cells as compared to the SAL-treated one [F (9.40)=10.42]; however, the fold change as observed is not as high as caspase-3 (**Figure 2E**). DACT showed a slight reduction in the LDH levels in PILO as well as the LPS+PILO model; however, no significant decrease was observed with RAPA and BUP in the combination model.

#### 3.1.4 BUP, DACT, and RAPA Inhibit the Nuclear Damage in PILO and LPS+PILO Induced HT22 Cells

Next, we tested the effects of BUP, DACT, and RAPA on the nuclear integrity in HT22 cells after treatment with both PILO and LPS+PILO. Herein, the propidium iodide stained cells exhibited increased nuclear damage in LPS, PILO, and LPS+PILO induced groups (F (9,40)=18.41) (**Figure 2F**). All three drugs, i.e., RAPA (p=0.0315), BUP (p=0.0026), and DACT (p=0.0041), attenuated nuclear damage in PILO; however, only BUP (p=0.0046) and DACT (p=0.0070) showed efficacy, but RAPA (p=0.2945) failed to reverse it in the LPS+PILO group. The cells were also observed under a fluorescence microscope, and images were captured for qualitative analysis (**Figure 2G**).

#### 3.1.5 Protein Levels Downstream of PI3K Activation by LPS+PILO Is Inhibited by BUP and DACT But Not RAPA

Our study showed the activation of both PI3K as well as ERK signaling events upon PILO and LPS treatment, which was further increased in the cells treated with the combination of LPS+PILO. BUP (p=0.0433) and DACT (p=0.0213), but not RAPA (p=0.997), decreased the Akt phosphorylation in the PILO model. Similar results were observed in BUP (p=0.0018), DACT (p=0.0016), and RAPA (p=0.2314) in the LPS+PILO model (F (9,60)=9.326) (**Figure 2H**). Further studies revealed an increase in p-pS670K/pS670K levels upon PILO and LPS+PILO groups post 24 h of exposure. Treatment with RAPA, BUP, and DACT decreased the levels in the PILO model; however, no significant decline was observed in RAPA-treated groups in the LPS+PILO model (**Figure 2I**). An increase in the pERK/ERK levels was observed upon treatment with all three drugs, out of which RAPA showed the least effect (**Figure 2J**).

#### 3.1.6 Phospho-NF-κB

An increase in the levels of phosphorylated nuclease factor-κ binding protein (NF-κB) was observed in PILO-treated groups, which was increased further on LPS priming (**Figure 2K**). DACT (p=0.218) and RAPA (p=0.274) decreased the NF-κB levels in the PILO model; however, BUP showed slight nonsignificant reduction. All three drugs attenuated the levels in the LPS+PILO (p=0.0094, p=0.0045, p=0.094) model.

#### 3.1.7 BUPA and DACT But Not RAPA Inhibits the Markers of Oxidative Stress in LPS+PILO Model

**Figure 3:**
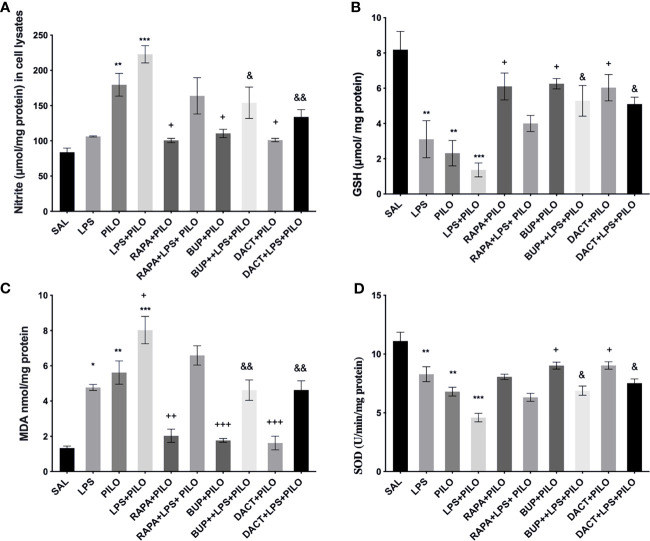
Effects of RAPA, BUP, and DACT on PILO versus LPS+PILO-induced HT-22 cells on **(A)** nitrite, **(B)** GSH, **(C)** MDA, and **(D)** SOD levels. Data are represented as mean ± SEM (n = 6) and analyzed by Tukey Kramer multiple comparison tests. The significance is ascertained as ***p < 0.001 *versus* SAL, **p < 0.01 *versus* SAL, *p < 0.05 *versus* SAL, ^+++^p < 0.01 *versus* PILO, ^++^p<0.01 *versus* PILO, ^+^p < 0.05 *versus* PILO, ^&&^p < 0.01 *versus* LPS+PILO, and ^&^p < 0.05 *versus* LPS+PILO.

Our *in vitro* results also demonstrated an increase in nitrite and MDA levels along with the reduction in GSH and SOD levels upon PILO and LPS+PILO treatment (**Figure 3**). All three drugs, except RAPA for SOD levels, showed antioxidant potential in cells treated with PILO. However, only BUP and DACT showed efficacy in the cells treated with the combination of LPS+PILO (**Figure 3**).

#### 3.1.8 BUP, DACT, and RAPA Modulates TGF-β Signaling in LPS+PILO Model

**Figure 4:**
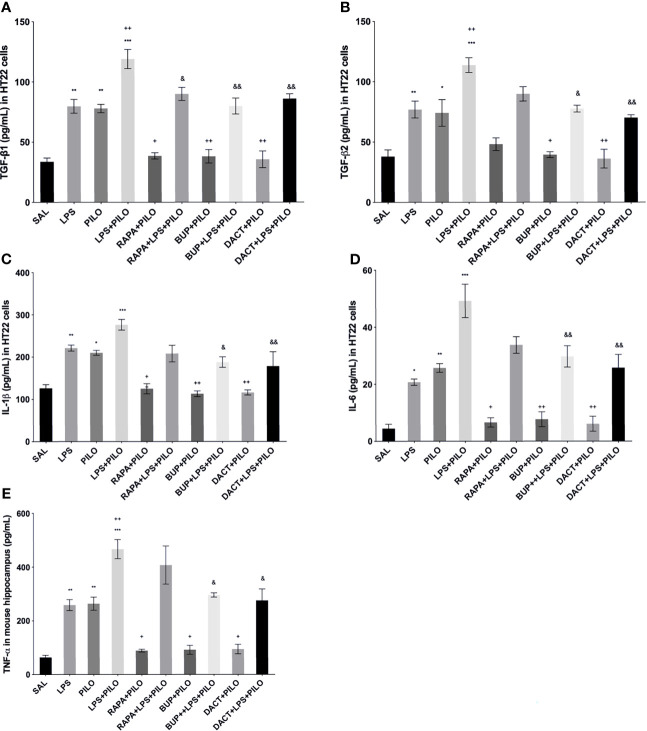
Effects of RAPA, BUP, and DACT on cytokine signaling in HT-22 cells. The cytokine levels (pg/ml) in the above figure were measured in cellular lysates. The figure is represented as effects on the levels of **(A)** TGF-β1, **(B)** TGF-β2, **(C)** IL-1β, **(D)** IL-6, and **(E)** TNF-α. The data are represented as mean ± SEM (n = 6) and analyzed by one-way ANOVA followed by Tukey Kramer multiple comparison tests. The significance is ascertained as ^***^p < 0.001 *versus* SAL, ^**^p<0.01 *versus* SAL, ^*^p<0.05 *versus* SAL, ^&&^p<0.001 *versus* LPS+PILO, ^&^p<0.05 *versus* LPS+PILO, ^++^p<0.01 *versus* PILO, and ^+^p<0.05 *versus* PILO.

The profound increase in both TGF-β1 and TGF-β2 was observed upon LPS priming as compared to PILO-treated HT22 cells. All three drugs reduced the TGF-β1 levels in both models *in vitro* [F (9,20)=29.27]; however, RAPA could not attenuate the levels of TGF-β2 in PILO as well as LPS+PILO models in HT22 cells [F (9,20)=17.07] (**Figure 4**). We also measured the third isoform of the TGF-β family, i.e., TGF-β3 in cellular lysates along with the *in vivo* brain and serum samples (data not shown). No change upon PILO induction was observed in both *in vitro* and *in vivo*; however, LPS+PILO resulted in a slight increase in TGF-β3 release in the cells, which was unaffected by drug treatment.

#### 3.1.9 PI3K Activity Acutely Modulates Cytokine Signaling in PILO *Versus* LPS+PILO Model in C57BL/6 Mice

LPS-, PILO-, as well as the LPS+PILO-treated cells showed a marked elevation in levels of TNF-α, IL-1β, and IL-6 as compared to saline with the combination showing the maximum elevation of cytokines. All three drugs attenuated the levels of these cytokines in PILO-treated cells. However, RAPA failed to attenuate the same upon LPS priming (**Figure 4**).

### 3.2 The *In Vivo* Studies

#### 3.2.1 Pharmacological Inhibition of PI3K and Dual PI3K/mTOR Activity Suppresses Neuroinflammation-Mediated Behavioral Seizures

**Figure 5:**
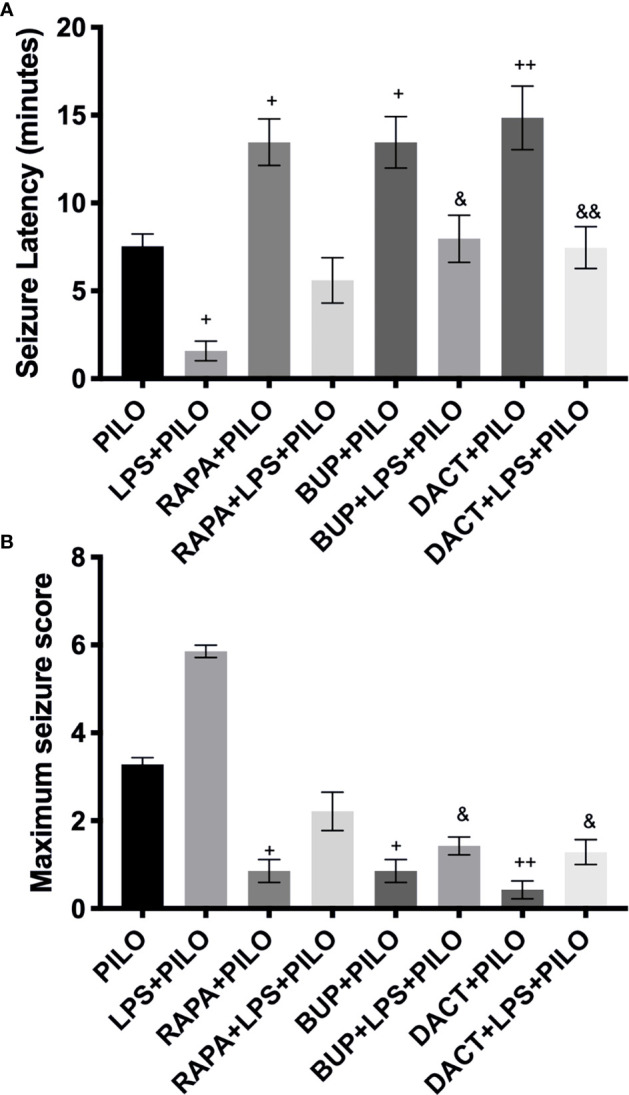
Effects of RAPA, BUP, and DACT on PILO *versus* LPS+PILO-induced seizures in male C57BL/6 mice. **(A)** Latency to seizures. The data are represented as mean ± SEM (n = 6) and analyzed by one-way ANOVA followed by Tukey–Kramer multiple comparison tests. **(B)** Seizure score. The data are represented as mean ± SEM (n = 6) and analyzed by Kruskal–Wallis analysis by ranks followed by Dunn’s multiple comparison tests. The significance is ascertained as ^++^p<0.01 *versus* PILO, ^+^p < 0.05 *versus* PILO, ^&&^p<0.01 *versus* LPS+PILO, and ^&^p<0.05 *versus* LPS+PILO.

It was observed that the low-grade seizures were induced within 7.54±0.70 and 1.588±0.55 min of PILO and LPS+PILO administration, respectively. In contrast with LPS+PILO induced seizures, tonic-clonic convulsions (stage 7) were not observed in PILO-alone groups, and most of the animals progressed to the stage of 4.5. BUP (p=0.0419) and DACT (p=0.0175) prolonged the latency and attenuated the seizure severity in both the models with DACT showing better protection (**Figure 5A**). However, RAPA failed to delay seizures in the LPS preconditioned (LPS+PILO) model [F (7,32) =13.01] (**Figure 5B**).

#### 3.2.2 PI3K and PI3K/mTOR, But Not mTOR Alone, Mitigates Seizure-Associated Pyknosis in Neuroinflammation-Mediated Seizures

**Figure 6:**
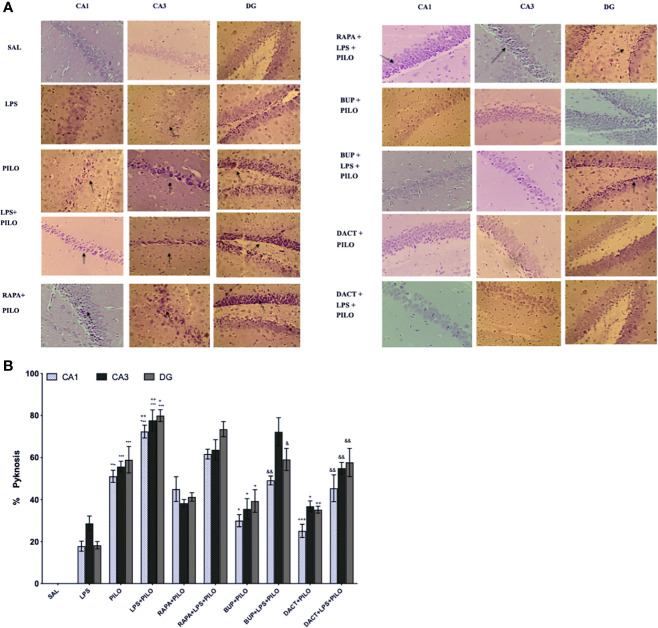
Effect of LPS priming 2 h prior to PILO exposure on neurodegeneration in C57BL/6 hippocampi. The photomicrographs represented in the above figure show the CV stained hippocampus sections, i.e., CA1, CA3, and DG regions under higher magnification (scale bar: 200 μm, n=3). The arrows represent significant pyknotic cells in those areas. The graph represents ^***^p<0.001 *versus* SAL, ^+++^p<0.001 *versus* PILO, ^++^p<0.01 *versus* PILO, ^+^p<0.05 *versus* PILO, ^&&^p<0.01 *versus* LPS+PILO, and ^&^p<0.05 *versus* LPS+PILO.

The saline-treated mice showed normal morphology (**Figure 6**), whereas PILO resulted in the significant pyknosis in the CA1, CA3, and dentate gyrus (DG) regions of the hippocampus, which was further potentiated by LPS priming in all three regions. Significant protection was observed with the 7-day prophylactic treatment of BUP and DACT in the PILO (p=0.022, p=0.0022) as well as LPS+PILO (p=0.0118, p=0.0053) groups. No neuronal protection was observed with RAPA in both models (**Figure 6**).

#### 3.2.3 PI3K/Akt Signaling Modulates the Inflammatory Signaling in Behavioral Seizures

##### 3.2 3.1.Phospho-Akt/Akt, Phospho-p70S6K/total-p70S6K, and Phospho-ERK/ERK

**Figure 7:**
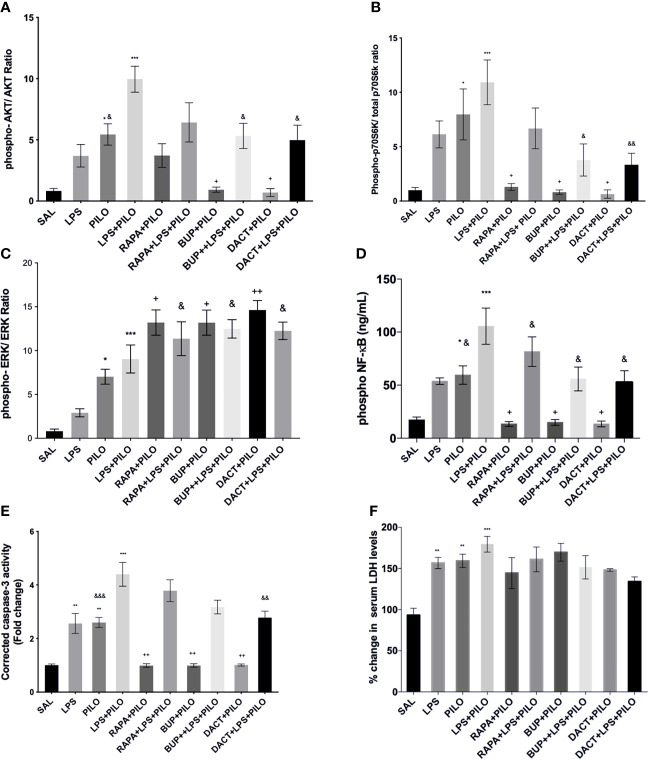
RAPA, BUP, and DACT alter PI3K pathway protein profile. Graphs show differential protein levels due to different treatments. **(A)** Hippocampal phospho-Akt/Akt, **(C)** hippocampal phospho-p70S6K/total-p70S6K, **(B)** hippocampal phospho-ERK/ERK, **(D)** hippocampal phospho-NF-κB levels (ng/mL), **(E)** corrected caspase-3 activity in mice hippocampus, and **(F)** serum LDH levels in C57BL/6 mice with or without LPS priming in the PILO model. Data are represented as mean ± SEM; n=3. The significance is ascertained as ***p<0.001 *versus* SAL, **p<0.01 *versus* SAL, ^*^p<0.05, ^++^p<0.01 *versus* PILO, ^+^p<0.05 *versus* PILO, ^&&^p<0.01 *versus* LPS+PILO, and ^&^p<0.05 *versus* LPS+PILO.

As shown in **Figure 7A**, we observed an elevation in pAkt/Akt ratios upon treatment with PILO. Significant elevation was observed upon LPS priming as compared to PILO alone. Upon drug treatments, BUP and DACT were able to reduce the levels of pAkt/Akt; however, RAPA failed to work in both PILO (p= 0.0402, p=0.0241, p=0.9530) and LPS+PILO (p=0.0309, p=0.0148, p=0.2217) models. Alongside, an elevation in the p-p70S6K/p70S6K and pERK/ERK ratios was also observed upon induction with PILO and LPS+PILO; however, p-p70S6K/p70S6K activity was reduced with all three drugs in the PILO model. RAPA could not decrease the levels in LPS+PILO induced animals (**Figure 7B**). Pretreatment with the test drugs resulted in further elevation of the same pERK/ERK in both models (**Figure 7C**).

##### 3.2.3.2 NF-κB

Downstream of this signaling, it was interesting to observe the profound increase in the levels of phosphorylated nuclease factor-κ binding protein (NF-κB) in PILO-treated groups, which was increased further on LPS priming (**Figure 7D**). All three drugs decreased the NF-κB levels in both models.

##### 3.2.3.3 Cleaved Caspase-3 and Serum LDH

On cleaved caspase-3, all three drugs, i.e., BUP (p=0.0030), DACT (p=0.0035), and RAPA (0.0030), showed a reduction in cleaved caspase-3 levels in the PILO model. However, only DACT (p=0.0027) reduced the levels in the combination (LPS+PILO) model [F (9,40)=24.01] (**Figure 7E**). As a marker of necrosis, we also tested the level of LDH in the mice serum. The test drugs could not attenuate the LDH levels in the serum in both models (**Figure 7F**).

##### 3.2.3.4 p53

**Figure 8:**
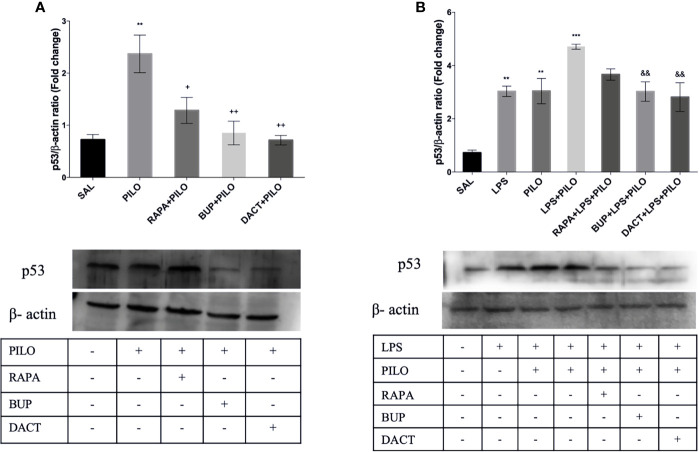
RAPA, BUP, and DACT alter PI3K pathway p53 levels in mice hippocampus. The figure represents protein levels due to different treatments. **(A)** Levels of p53 in PILO and **(B)** levels of p53 in the LPS-primed PILO model. Treatment groups are indicated in the table against the band in the figure. Densitometry values for p53 were normalized to β-actin and expressed as fold change relative to the control group. The data are represented as mean ± SEM (n = 3) and analyzed by one-way ANOVA followed by Tukey Kramer multiple comparison tests. The significance is ascertained as ^***^p < 0.001 *versus* SAL, ^**^p<0.01 *versus* SAL, ^&&^p<0.001 *versus* LPS+PILO, ^++^p<0.01 *versus* PILO, and ^+^p<0.05 *versus* PILO.

As shown in **Figure 8**, p53 is upregulated in PILO [F (4,10)=9.638] (**Figure 8A**) as well as in LPS+PILO [F (6,14)=13.23] (**Figure 8B**) mediated cell death, which is further inhibited by RAPA (p=0.453), BUP (p=0.0059), and DACT (p=0.0032) in the PILO model, and DACT further inhibited DACT (p=0.0151) and BUP (p=0.034), but not RAPA, in the LPS+PILO model.

##### 3.2.3.5 Oxidative Stress

**Figure 9:**
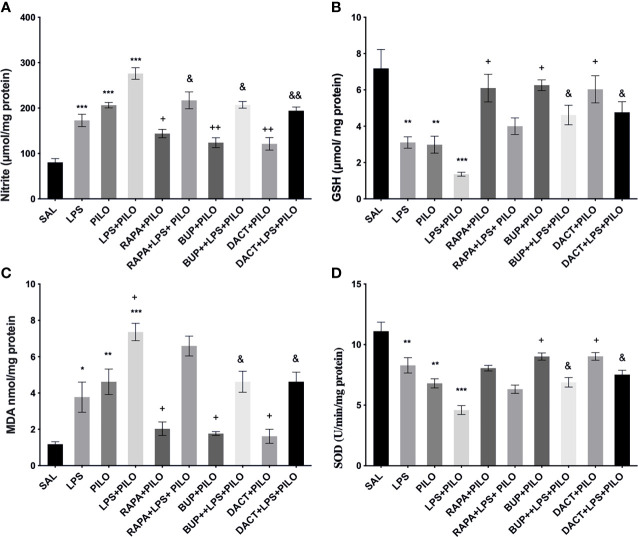
Effects of RAPA, BUP, and DACT on PILO *versus* LPS+PILO-induced on oxidative stress levels in C57BL/6 mice. **(A)** Nitrite, **(B)** GSH, **(C)** MDA, and **(D)** SOD levels. Data are represented as mean ± SEM (n=3) and analyzed by Tukey Kramer multiple comparison tests. The significance is ascertained as ***p < 0.001 *versus* SAL, **p < 0.01 *versus* SAL, *p < 0.05 *versus* SAL, ^++^p < 0.01 *versus* PILO, ^+^p < 0.05 *versus* PILO, ^&&^p < 0.01 *versus* LPS+PILO, and ^&^p < 0.05 *versus* LPS+PILO.

Our results also demonstrated an increase in nitrite and MDA levels along with the reduction in GSH and SOD levels upon PILO and LPS+PILO treatment (**Figure 9**). All three drugs, except RAPA for SOD levels, showed antioxidant potential in the PILO model. However, only BUP and DACT showed efficacy in the combination model (**Figure 9**).

#### 3.2.4 PI3K Activity Acutely Modulates Cytokine Signaling in PILO *Versus* LPS+PILO Model in C57BL/6 Mice

##### 3.2.4.1 Effect on TGF-β Signaling

PILO+LPS administration in mice showed a significant increase in hippocampal TGFβ1 and TGFβ2 levels as compared to PILO alone. BUP and DACT decreased the levels of β1 in both PILO (p=0.0094, p=0.0050) and LPS+PILO (p=0.0034, p=0.0013) models; however, RAPA (p=0.0425) was only effective in the PILO model (F (9,20)=20.38).

**Figure 10:**
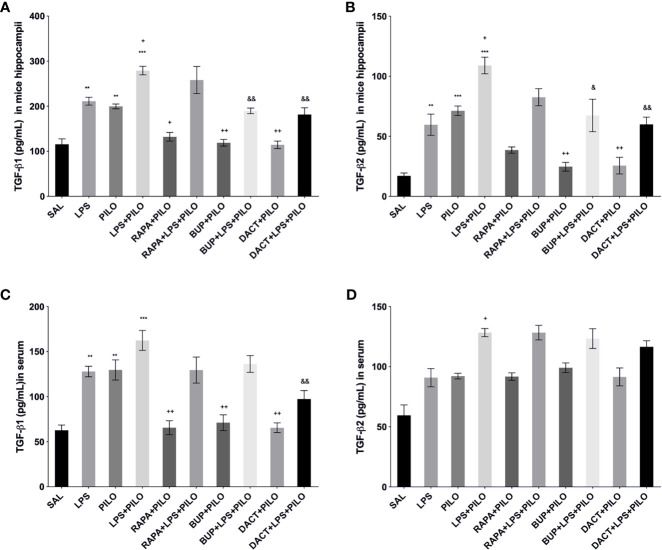
Effects of RAPA, BUP, and DACT on TGF-β signaling in LPS/PILO-treated C57BL/6 mice. The cytokine levels (pg/ml) in the above figure were measured hippocampal and serum samples of C57BL/6 mice. The figure is represented as effects on the levels of TGF-β1 in **(A)** hippocampal and **(B)** serum samples. Effects on the levels of TGF-β2 in **(c)** hippocampal and **(d)** serum samples. The data are represented as mean ± SEM (n=3) and analyzed by one-way ANOVA followed by Tukey Kramer multiple comparison tests. The significance is ascertained as ^***^p < 0.001 *versus* SAL, ^**^p<0.01 *versus* SAL, ^&&^p<0.001 *versus* LPS+PILO, ^&^p<0.05 *versus* LPS+PILO, ^++^p<0.01 *versus* PILO, and ^+^p<0.05 *versus* PILO.

TGFβ2 was reduced by BUP and DACT (p=0.0041, p=0.0050) in PILO as well as LPS+PILO (p=0.0122, p=0.0024) models; however, RAPA failed to attenuate the levels of both (F (9, 20)=17.37). An increase in the levels of TGF-β1 in the serum of mice was also observed; however, no statistically significant differences in the release were observed between PILO and LPS+PILO groups. PILO-induced wells showed reduction in TGF-β1 levels by all three drugs; however, no mitigation in serum levels (except TGF-β1 by DACT) was observed in the combination model. No attenuation in the serum TGF-β2 level was observed by all three drugs in both models (**Figure 10**).

##### 3.2.4.2 Effects on TNF-α, IL-1β, and IL-6 Levels

**Figure 11:**
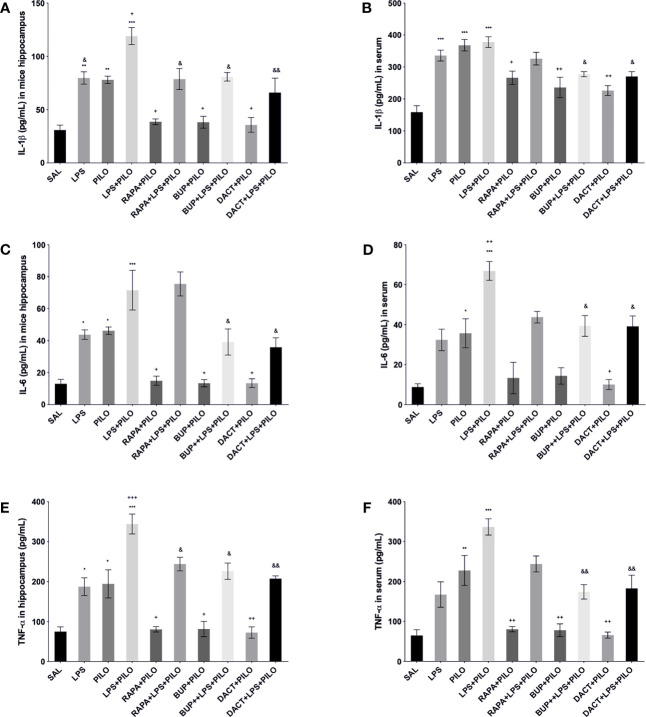
Effects of the RAPA, BUP, and DACT on TNF-α, IL-1β, and IL-6 levels (pg/ml) in LPS/PILO-treated C57BL/6 mice. The cytokine levels (pg/ml) in the above figure were measured in the *in vivo* hippocampal and serum samples of C57BL/6 mice. The figure is represented as effects on the levels of IL-6 **(A)** *in vivo* hippocampal samples and **(B)** serum samples. Effects on IL-1β levels in **(C)** *in vivo* hippocampal samples and **(D)** serum samples. Effects on the TNF-α levels in **(E)** *in vivo* hippocampal samples and **(F)** serum samples. The data are represented as mean ± SEM (n = 3) and analyzed by one-way ANOVA followed by Tukey Kramer multiple comparison tests. ^***^p< 0.001 *versus* SAL, ^**^p<0.01 *versus* SAL, ^*^p<0.05 *versus* SAL, ^&&^p<0.01 *versus* LPS+PILO, ^&^p<0.05 *versus* LPS+PILO, ^++^p<0.01 *versus* PILO, and ^+^p<0.05 *versus* PILO.

All three drugs attenuated the levels of hippocampal TNF-α, IL-1β, and IL-6 in the PILO model. Furthermore, to understand the comparative peripheral effects of the test drugs in both models, the serum levels were also measured that revealed the increased serum cytokine levels in LPS, PILO, as well as LPS+PILO induced animals. Significant differences in the induction levels were observed in LPS, PILO, and LPS+PILO groups; the drugs showed variable effects and mostly reversed the enhanced serum cytokine levels in both models. However, RAPA could not reverse both hippocampal and serum IL-6 levels in the combination model (**Figure 11**).

#### 3.2.5 PI3K Activity Affects Neurobehavioral Changes Associated With Seizures in C57BL/6 Mice

##### 3.2.5.1 Morris Water Maze

**Figure 12:**
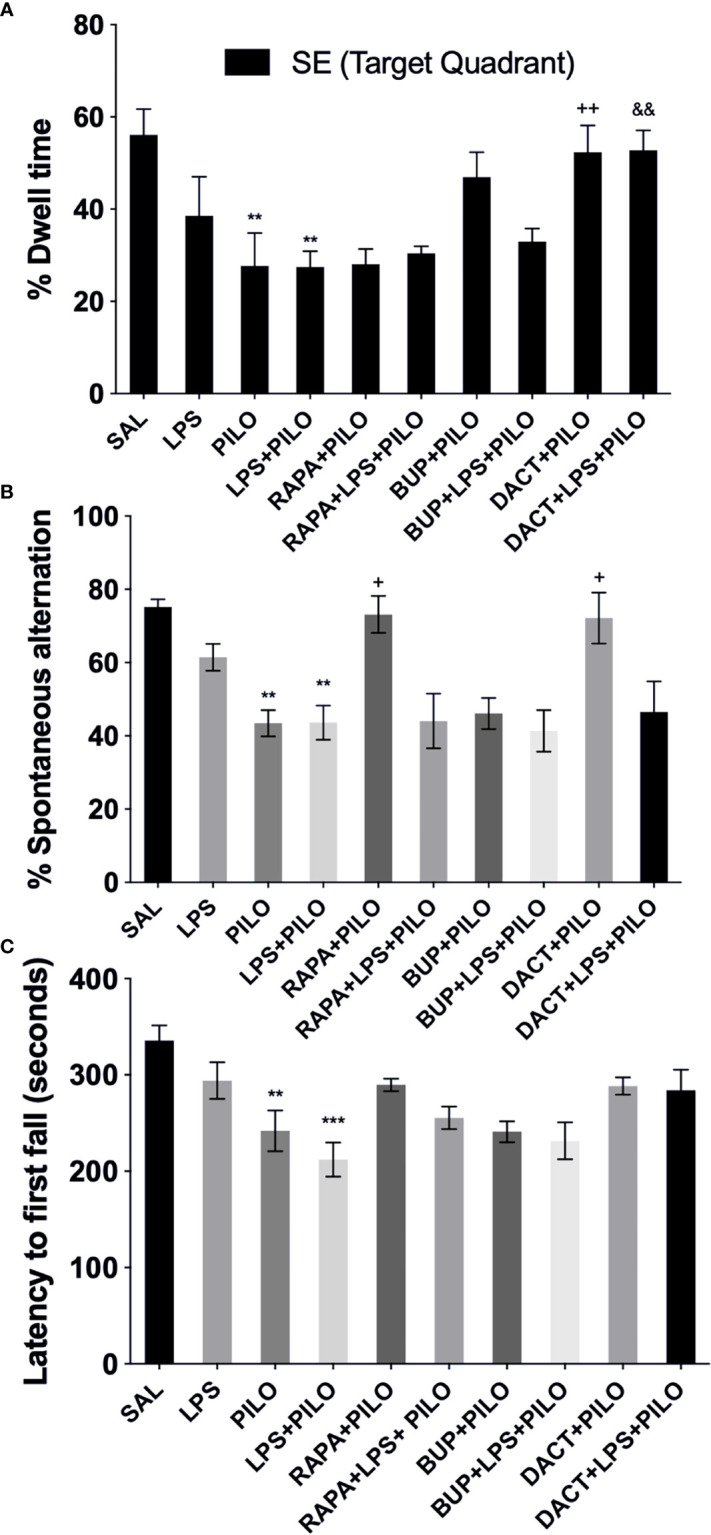
Effects of RAPA, BUP, and DACT on cognitive and motor functions in LPS/PILO-treated C57BL/6 mice. **(A)** % dwell time in the reference memory test, **(B)** motor coordination, and **(C)** % spontaneous alternation. The data are represented as mean ± SEM (n = 5) and analyzed by one-way ANOVA followed by Tukey Kramer multiple comparison tests. ^***^p<0.001 *versus* SAL, **p < 0.01 *versus* SAL, ^&&^p < 0.001 *versus* LPS+PILO, ^++^p < 0.01 *versus* PILO, ^+^p < 0.05 *versus* LPS+PILO.

*% Quadrant dwell time*: After the 5-day training protocol, the animals were subjected to PILO and/or LPS+PILO induced seizures; the platform was removed, and a probe trial was conducted 24 h after the previous acquisition trial. The percentage of time spent in the target quadrant (southeast, SE quadrant) was measured. As shown in **Figure 12A**, PILO- and LPS+PILO-treated animals showed a decrease in dwell time in comparison to saline-treated mice. Only DACT could increase the dwell time in both PILO (p=0.009) and LPS+PILO (p=0.0073) models. BUP showed a nonsignificant increase in dwell time in the PILO model; however, it could not improve cognitive activity in the combination model (**Figure 12A**).

##### 3.2.5.2 Spontaneous Alternation Behavior

Post PILO- and/LPS+PILO-induced seizures, the animals showed a significant decrease in the % alternation as compared to the saline-treated ones. As shown in **Figure 12B**, the 7-day prophylactic treatment of DACT (0.0202) as well as RAPA (0.0191) displayed improved alternation behavior in the PILO model only; however, BUP displayed no improvement in both models and could not return the values to the baseline (as compared to saline) (**Figure 12B**).

##### 3.2.5.3 Motor Coordination

The latency to the first fall for the PILO *versus* LPS+PILO model is represented in **Figure 12C** after 24 h of seizure induction. Latency to the first fall was reduced upon PILO (P=0.0061) and LPS+PILO (p=0.0001) induction; however, no increase in latency was observed upon drug treatment in both models.

## Discussion

Phosphoinositide-3-kinase (PI3K), a membrane signaling kinase, is overactivated in rodents in inflammatory and epileptic conditions. Herein, we demonstrate that neuroinflammation-mediated seizures are associated with apoptotic neuronal cell death and degeneration associated with deregulated PI3K/Akt/mTOR signaling. By suppressing this pathway using PI3K and PI3K/mTOR dual pharmacological inhibitors, we were able to see reduction in PILO as well as LPS+PILO induced seizures along with apoptotic cell death and neurodegeneration mediated by the hyperactivated PI3K/Akt pathway. The mTOR inhibition alone could not attenuate the seizures induced by the combination of LPS and PILO. Hence, our results revealed the plausible association of the PI3K pathway in seizures and seizure-induced neuronal death, which has potential clinical relevance. To our knowledge, this is the first study to evaluate the comparative effects of the pan-PI3K inhibitor, BUP and PI3K/mTOR inhibitor, and DACT on neuronal inflammation-mediated seizures and cell death in both PILO as well as LPS primed PILO models.

In our work, we first validated the involvement of PI3K/Akt molecular mechanism using an *in vitro* model of neuronal inflammation optimized in our laboratory using mouse hippocampal HT22 cells. We tried to understand the possible underlying cell signaling mechanisms involved and optimized the cell-based assay in HT22 cells using various concentrations of the inducers and drugs, i.e., LPS (0.1–10 μg/ml), PILO (5–500 μM), and their combinations (LPS+PILO). Subsequently, to evaluate the cell death induced by the test drugs themselves in 24 h, we treated the cultures with various concentrations of BUP (25–520 nM), DACT (5–100 nM), and RAPA (0.1–5 nM) to screen and test their activities in the presence and absence of the inducers (optimization data not shown). These concentration ranges were selected based on the available data (45–47). In our laboratory conditions, the IC50 of drugs, i.e., BUP (50 nM), DACT (15 nM), and RAPA (4 nM), and the EC50 of the inducers PILO (200 μM) and LPS (1 μg/ml) were observed and showed desired induction/survival in HT22 cells (data not shown). We next explored the pattern of neuronal death at the molecular level and measured the neuronal survival, levels of caspase-3, LDH leakage, nuclear integrity, and mitochondrial membrane potential in drug-treated and nontreated HT-22 cells (48–50).

One of the earliest changes that occur in at the time of neurodegeneration and cell death is the change in MMP (51), and therefore, we tested the effects on mitochondrial membrane potential using rhodamine 1,2,3 dye in the two models. The increased level of ΔΨm upon PILO and LPS+PILO exposure, as observed in our work, is indicative of the decreased cell viability and can be correlated with seizure pathology. In line with our work, accumulating evidences also support the association of mitochondrial dysfunction and oxidative stress in the pathophysiology of seizures (52). Propidium iodide staining also showed increased nuclear damage in PILO and LPS+PILO groups, which was attenuated by all three drugs in the PILO model and by BUP and DACT in the combination model. Irrespective of the above, MMP alone cannot be considered a specific marker for apoptosis and neuronal death, and therefore, we further tested the involvement of apoptotic and necrotic pathways in PILO and LPS+PILO induced cells by measuring cleaved caspase-3 and LDH (% leakage), the markers for apoptotic and necrotic cell death, respectively, in neuronal cultures (48–50). Caspase-3 is suggested as a major mediator of neuronal apoptosis, which is associated with both seizures (53) as well as inflammatory events (54). On the other hand, %LDH leakage from the neuronal cells is considered as one of the main markers of necrotic cell death (55). Our results showed the major contribution of apoptotic events in correlation with the increased levels of caspase-3 in neuronal cultures. A previous report has also suggested the activation of the caspase-3 pathway in the injured neurons post-24 h following seizures, which correlates with our study (56). All three drugs showed a reduction in cleaved caspase-3 levels in the PILO model. However, the PI3K/mTOR dual inhibition by DACT showed the highest efficacy in PILO as well as in the combination model as compared to PI3K or mTOR inhibition *per se.* It validates our other findings on the altered levels of the markers regulating the cell death and survival in seizures and inflammation and shows the correlation of PI3Ks with the same.

Because seizures are associated with cellular damage and death, we further proceeded with the *in vivo* studies, in which we observed the reduction in seizure latency and an increase in the number of seizure episodes upon LPS priming. In previous studies too, LPS priming enhanced the seizure susceptibility to glutaric (57) as well as kainic acid (subconvulsive doses) in rat models (58). All three drugs, i.e., BUP, DACT, and RAPA, delayed the seizures and mitigated the seizure severity in PILO-induced seizures; however, RAPA was not found to be effective in delaying the onset to seizures in the LPS preconditioned model, which indicates that mTOR inhibition alone cannot protect against neuroinflammation-mediated seizures. In line with our findings, Roy et al. (8) also demonstrated the abnormal PI3K activity in the development of seizures associated with brain overgrowth disorders and suggested the potential of the PI3K inhibitor BUP in the treatment of seizures in pediatric patients of such brain overgrowth disorders (8). Similarly, recent studies have also delineated the involvement of this pathway in a hippocampal culture model (7, 59). When comparing the efficacies, we observed that DACT showed better protection, which might be due to effective inhibition of PI3K as well as mTORC1/2 at two nodes simultaneously.

The results on seizures correlated well with the histopathological analysis of the *in vivo* brain samples and pyknosis in CA1, CA3, and dentate gyrus (DG) regions of the hippocampus upon PILO exposure and its potentiation by LPS priming. Here, too, PI3K and PI3K/mTOR inhibitors protected against neurodegenerative changes in both models demonstrating the contribution of this pathway in the seizure-induced neuronal pyknosis. Previous studies have also suggested the involvement of PI3K/Akt related pathways in the cell death and survival related decisions of the neuronal cells (60, 61). Next, in the Morris maze test, we observed impairment of cognitive activity post-seizures induced by PILO and LPS priming. Protection against PILO and LPS+PILO-induced cognitive impairment was observed by DACT but not by BUP. This might be again due to the effective inhibition of the pathway at two nodes. However, only RAPA and DACT showed protection in SAB test. In line with our work, Bellozi and co-workers (62) have also shown the neuroprotective ability of DACT in amyloid-β1–42 induced memory impairment and neuronal death (62). None of the drugs, however, showed any effect on motor impairment in the rota-rod test.

We further tried to probe the pattern of the downstream signaling events following PI3K/Akt activation subsequent to PILO- and LPS+PILO-induced seizures and associated neurodegenerative pathology. Being a biomarker for the activation of PI3K signaling, the levels of pAkt/Akt were measured, and we observed the reduced pAkt/Akt ratios upon BUP and DACT administration. Previous literature also points toward the hyperactivation of PI3K/Akt/mTOR signaling within 1 h of seizures. Furthermore, it has been reported that PI3K and mTOR inhibitors, LY294002 or RAPA, respectively, reduce the pAkt expression induced by IL-1β in brain cells (63). Also, the activation of the p70S6 kinase activity is an alternate marker for the activation of the mTOR pathway, which further promotes the translation of cell death and survival related proteins crucial for seizure-associated neurodegeneration and apoptosis. Existing literature points toward the essential role of this PI3K/mTORC1/S6K pathway in cell death and apoptosis-related pathways (5, 64, 65), which was substantiated in our study using the PILO and LPS+PILO models. An increase in phosphorylation of p70S6 kinase was observed upon LPS/PILO induction, which suggested the overactivation of this pathway *via* mTOR. This increased hippocampal phospho-p70S6 kinase/total p70S6 kinase ratio was reduced by RAPA, BUP, and DACT in the PILO model. However, in the LPS+PILO model, RAPA (mTORC1 and FRAP inhibitor) could not reduce Akt as well as p70S6 kinase activity possibly due to the reactivation of the pathway by functionally different complex of mTOR, i.e., mTORC2. mTORC1 is well characterized for its structure, regulation, and function in the last decade and is inhibited by RAPA at the doses used in our study. DACT, an inhibitor of both mTORC1 and mTORC2 (along with PI3K), reduced p70S6K kinase activity in both the models *in vitro* and *in vivo*. This could be the plausible reason for partial RAPA resistance observed in the combination model, where overactivation of the inflammatory signaling was also observed. Existing literature also demonstrated that only chronic administration of RAPA can substantially inhibit mTORC2 (66, 67), which could be the possible reason for its inefficacy at lower doses used in our work. Previous studies have also demonstrated an increase in S6K activity in hippocampus post-seizures (5, 64). Zeng and coworkers (13) showed the return of this increased S6K activity to the baseline by 24 h followed by a second peak period starting from the third day of kainic acid treatment (13, 68). In contrast, we observed increased p70S6K activity both *in vitro* and *in vivo* post 24 h of LPS/PILO exposure. These differences might be due to the fact that they have used a kainate model, whereas we have employed a different one.

Next, considering the PI3K-ERK crosstalk and compensation, we tested if the treatments with these drugs also affect the levels of the pERK/ERK ratio. Our study suggested the cross-activation of the ERK pathway post-LPS as well as PILO-induced inflammation and seizures, which is in line with previous studies (61, 69). The pharmacological blockade of the PI3K/Akt/mTOR pathway using BUP, DACT, and RAPA resulted in the overactivation of pERK/ERK levels. A previous study in the breast cancer cells has also shown overactivation of ERK signaling upon PI3K inhibition (70). Our work also indicates the activation of ERK-dependent compensatory pathway upon the PI3K and PI3K/mTOR inhibition. In clinical perspective, it would be interesting to evaluate the effects of the combination therapy of PI3K and PI3K/mTOR inhibitors with ERK inhibitors in preclinical models in the future.

The dependency of the prolonged seizures on the cleaved caspase-3 levels has been reported previously (71). We observed an increase in the same post-24 h of seizure induction. Another study has also suggested the activation of the caspase-3 pathway in the injured neurons post-24 h following seizures, which correlates with our work (56). Furthermore, in line with the behavioral findings, all three drugs demonstrated reduction in cleaved caspase-3 levels in the PILO model. However, only DACT reduced the levels in the combination model. Activation of caspase-3 in neuronal cells with damaged DNA is also associated with the p53 pathway in excitotoxic neuronal cell death and neurodegeneration due to *status epilepticus* (72). The latter is also known to be upregulated in the hippocampal samples of patients with intractable TLE (73). We also observed that the PI3K-dependent phosphorylation of Akt/Caspase-3 regulates the p53 expression as demonstrated in hippocampal lysate, through western immunoblotting, to be upregulated in PILO as well as LPS+PILO mediated cell death, further inhibited by DACT and BUP, but not so much by RAPA. More recently, the neuronal availability of the enzyme LDH and its serum levels is being correlated with the susceptibility to the epileptic seizures (74). This enzyme catalyzes the interconversion of lactate and pyruvate, and its inhibition can effectively reduce the excitability of the neurons. LDH inhibition has already been studied for the suppression of seizures (75). Our results also showed an elevation in serum LDH levels upon seizure induction, but the test drugs had no effect on LDH levels in both models. Hence, our study supports the major association of caspase-3/p53-related apoptotic pathway instead of LDH-associated necrotic events downstream of PI3K in the behavioral seizures and neurodegenerative changes.

Seizures are associated with the cellular redox and aging (76). In line with this, an increase in oxidative stress levels was observed upon both PILO as well as LPS+PILO induction. Test drugs showed antioxidative potential in the PILO model, except RAPA for SOD levels. However, PI3K and PI3K/mTOR dual inhibition by BUP and DACT, respectively, showed efficacy in the combination model. This shows that the PI3K/Akt signaling and caspase-3/p53 activation are directly linked with oxidative stress. Caspase-3 and p53 activity has already been shown to be associated with increased oxidative stress and injury in the brain (77–79). In addition, previous studies have demonstrated the involvement of the PI3K/Akt pathway in diazoxide-induced oxidative injury in the brain (80). The authors also showed the reduction in MDA and increment in SOD levels upon treatment with wortmannin, which is a pan-PI3K inhibitor.

We also observed a profound increase in the levels of phosphorylated nuclease factor-κ binding protein (NF-κB) upon PILO as well as LPS+PILO administration, which is a marker for inflammation, and was found to be associated with the high levels of oxidative stress induced by PILO and LPS+PILO upon PI3K/Akt activation. All three drugs decreased the NF-κB levels in both models. In another report, reduction in PILO-induced cerebral cortex inflammation in epileptic rats was observed by inhibiting NF-κB, which shows the association of the increased NF- κB levels and inflammation-mediated seizures (81). The induction of NF-κB further triggers the transcription of various proinflammatory genes encoding cytokines, which ultimately result in the release of various cytokines. Clinical studies also highlight the increase in the levels of proinflammatory cytokines in neurons and glia in the brain tissue obtained from patients of TLE and drug-resistant epilepsies (82–85). For preclinical studies too, the release of IL-1β, IL-6, and TNF-α is considered to be crucial for tonic-clonic seizures (86), and therefore, we explored the pattern of IL-1β, IL-6, TNF-α, and TGF-β signaling in both the brain and serum and observed the involvement of cytokine signaling downstream of the PI3K/Akt pathway in seizures. In our work, PI3K and PI3K/mTOR inhibition was found to be crucial for the cytokine release pattern in the murine brain. Our findings are in agreement with the previous works suggesting the dependency of cytokine signaling on the PI3K pathway in the brain (87, 88). Notably, our findings do not rule out the association of the mTOR-mediated pathway in this phenomenon. mTOR, however involved, might not be the only major pathway. RAPA and its analogues provide only incomplete inhibition of mTOR (89). DACT competitively inhibits PI3K class 1 along with mTOR C1/2 kinase activities attenuating the PI3K reactivation and mTORC2 Akt reactivation (90, 91). RAPA resistance in this model might be associated with the reactivation of the pathway by mTORC2 activity. In contrast, another study associates RAPA to be associated with the regulation of mTORC2 activity in glioblastoma cells (92); however, its effect in seizures requires further elucidation. Our *in vitro* and *in vivo* studies showed higher efficacy of DACT as compared to BUP and RAPA in the PILO as well as LPS+PILO model, which supports the stronger antiseizure effects of DACT as compared to the other two drugs.

## 5 Conclusion

**Figure 13:**
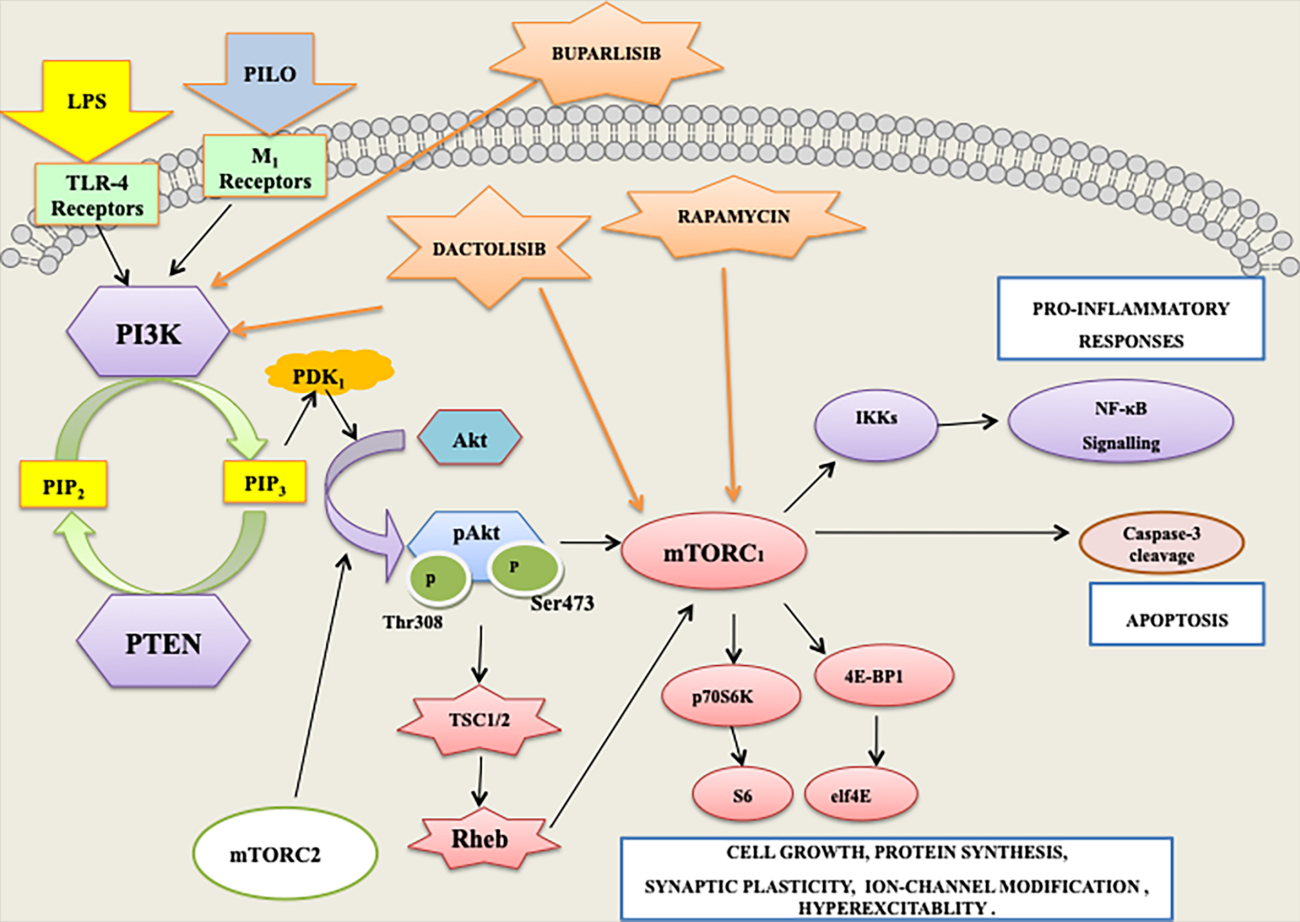
PI3K/AKT/mTOR/p70S6K signaling mediates the apoptotic and inflammatory responses associated with lipopolysaccharide (LPS) and pilocarpine (PILO) induced seizures.

Our study suggested the plausible involvement of PI3K/Akt/p70S6K and p53 mediated apoptotic and inflammatory pathways in the neuroinflammation-mediated seizures and associated neurodegenerative changes, which showed that the dual inhibition of this pathway by DACT can be tried as a promising treatment therapy in the future (**Figure 13**). We also observed that mTOR may indeed act *via* indirect means through the activation of other kinases that, in turn, regulate neuroinflammation-mediated seizures; however, proper elucidation of this pathway remains. To the best of our knowledge, this study is the first of its kind to explore the efficacy of these compounds in the PILO and LPS+PILO induced models in HT-22 cells and C57BL/6 mice. However, it mandates further studies for better insight of this pathway as molecular targets in neuronal inflammation-mediated seizures and neurodegeneration, which is still required to support and ease their clinical use. This may aid in the development of successful and broadly applicable interventions to treat seizures associated with systemic inflammatory diseases, which, nevertheless, stay as the primary unmet need in the present therapy.

## Data Availability Statement

The original contributions presented in the study are included in the article/**Supplementary Material**. Further inquiries can be directed to the corresponding author.

## Ethics Statement

All the experiments were carried out in strict accordance with CPCSEA (Committee for the Purpose of Control and Supervision of Experiments on Animals, Ministry of Environment and Forests, Govt. of India) guidelines and the in-house guidelines of the Institutional Animal Ethics Committee of Jamia Hamdard, New Delhi, India (Registration no. JH/993/CPCSEA) under a protocol approved (Protocol no. 1312, 2017) by the committee.

## Author Contributions

PV, RT, and DV conceived and designed the study. DV prepared the basic hypothesis and conceptualized the work. PV performed the experiments. RT guided PV for the cell-culture part of the work. PV and DV participated in discussions and manuscript preparation. All authors contributed to the article and approved the submitted version.

## Funding

This work was supported by the Department of Science and Technology (DST), Government of India [DST-INSPIRE-Fellowship, IF160142, 2016]. We thank Aicte Modrob grant for supporting a part of the work.

## Conflict of Interest

The authors declare that the research was conducted in the absence of any commercial or financial relationships that could be construed as a potential conflict of interest.

## Publisher’s Note

All claims expressed in this article are solely those of the authors and do not necessarily represent those of their affiliated organizations, or those of the publisher, the editors and the reviewers. Any product that may be evaluated in this article, or claim that may be made by its manufacturer, is not guaranteed or endorsed by the publisher.

## Supplementary Material

Supplementary File 1 Uncropped gel image for levels of p53 in hippocampus of PILO induced C57BL/6 mice.

Supplementary File 2 Uncropped gel image for levels of β-actin in hippocampus of PILO induced C57BL/6 mice.

Supplementary File 3 Uncropped gel image for levels of p53 in hippocampus of LPS-primed PILO induced C57BL/6 mice.

Supplementary File 4 Uncropped gel image for levels of β-actin in hippocampus of LPS-primed PILO induced C57BL/6 mice

Supplementary File 5 List of reagents, drugs and commercially available ELISA kids and antibodies.
