# Supplementary figures and images for: Dual Targeting by Inhibition of Phosphoinositide-3-Kinase and Mammalian Target of Rapamycin Attenuates the Neuroinflammatory Responses in Murine Hippocampal Cells and Seizures in C57BL/6 Mice
Source: Front Immunol. 2021 Nov 23;12:739452. doi: 10.3389/fimmu.2021.739452 (PMC8650161; doi:10.3389/fimmu.2021.739452)

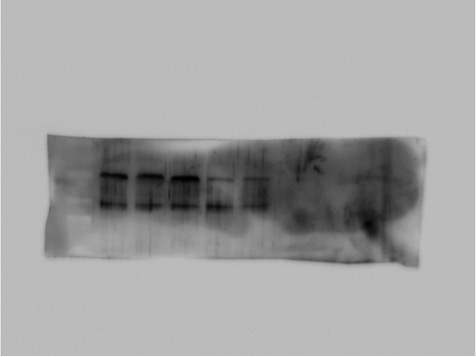

Supplement: Supplementary File 1 — Uncropped gel image for levels of p53 in hippocampus of PILO induced C57BL/6 mice. [file Image_1.tiff]

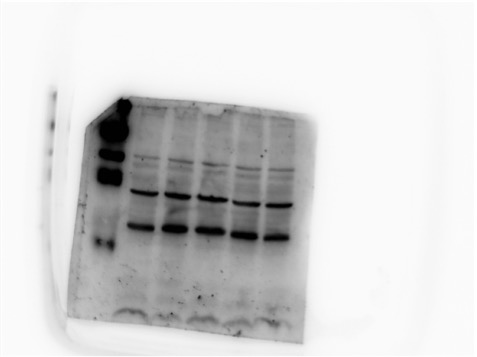

Supplement: Supplementary File 2 — Uncropped gel image for levels of β-actin in hippocampus of PILO induced C57BL/6 mice. [file Image_2.tiff]

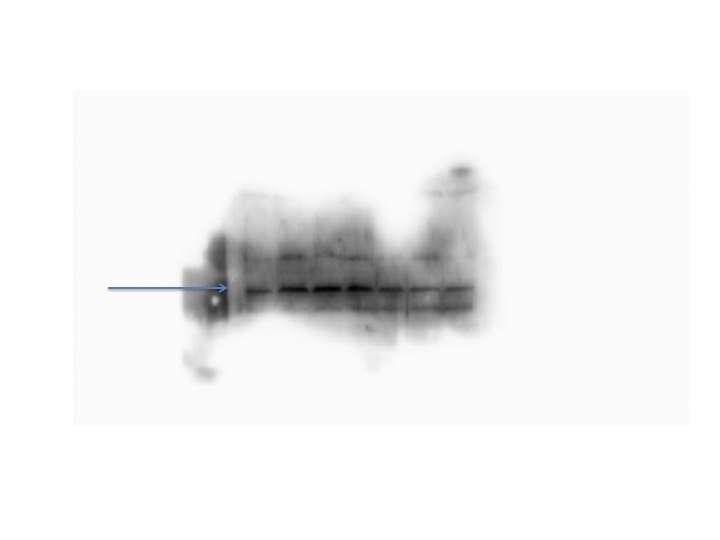

Supplement: Supplementary File 3 — Uncropped gel image for levels of p53 in hippocampus of LPS-primed PILO induced C57BL/6 mice. [file Image_3.tiff]

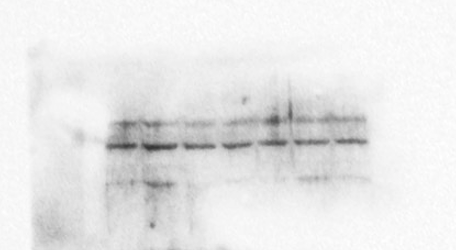

Supplement: Supplementary File 4 — Uncropped gel image for levels of β-actin in hippocampus of LPS-primed PILO induced C57BL/6 mice [file Image_4.tif]
