# Supplementary material for: Dual Targeting by Inhibition of Phosphoinositide-3-Kinase and Mammalian Target of Rapamycin Attenuates the Neuroinflammatory Responses in Murine Hippocampal Cells and Seizures in C57BL/6 Mice
Source: Front Immunol. 2021 Nov 23;12:739452. doi: 10.3389/fimmu.2021.739452 (PMC8650161; doi:10.3389/fimmu.2021.739452)
Supplement: Supplementary File 5 — List of reagents, drugs and commercially available ELISA kids and antibodies. [file DataSheet_5.docx]

Supplementary file 5

### **Drugs and Chemicals:**

| **Drugs/Chemicals** | **Company** | |
| --- | --- | --- |
| 2′,7′-Dichlorofluorescin diacetate (DCFDA) | Sigma Aldrich |  |
| Acetic acid | Sigma Aldrich |  |
| Acrylamide | Sigma Aldrich |  |
| Ammonium per sulphate (APS) | Sigma Aldrich |  |
| Antimycotic-antibiotic | Himedia |  |
| Bis-Acrylamide | Thermo scientific |  |
| Bovine serum albumin (BSA) | Sigma-Aldrich |  |
| Bradford reagent | Sigma-Aldrich |  |
| Bromophenol blue | Sigma-Aldrich |  |
| Buparlisib (NVP-BKM-120) | Cayman Chemicals |  |
| Carbamazepine | Sigma-Aldrich |  |
| Chemiluminescent and chemifluorescent HRP substrate | Pierce, Thermo Scientific |  |
| Coomassie blue | Thermo Scientific |  |
| CuSO_4_ (Copper Sulphate) | SDFCL, India |  |
| Dactolisib | Cayman Chemicals |  |
| Diazepam Injection | Neon Laboratories Ltd |  |
| Diethylether | Sigma Aldrich |  |
| Disodium hydrogen phosphate  (Na2HPO_4_) | Sigma Aldrich |  |
| DMEM-High glucose media | Sigma Aldrich |  |
| DMSO | Sigma Aldrich |  |
| DTNB (5,5-Dithiobis(2-nitrobenzoic acid)) | SRL, India |  |
| DTT | Sigma Aldrich |  |
| FBS | Himedia |  |
| Folin and ciocalteu’s phenol reagent | SRL, India |  |
| Formaldehyde | SRL diagnostics |  |
| Glycerol | Sigma Aldrich |  |
| Glycine | Sigma Aldrich |  |
| Hydrochloric acid | Sigma Aldrich |  |
| Isopropyl alcohol | Sigma Aldrich |  |
| Lipopolysaccharide  (*Escherichia coli*O111:B4) | Sigma-Aldrich |  |
| Methanol | Sigma Aldrich |  |
| Methyl Scopolamine | Neon Laboratories |  |
| n-butanol | SRL, India |  |
| Na_2_CO_3_ (Sodium Carbonate) | SDFCL, India |  |
| NP-40 | Sigma Aldrich |  |
| PI Cocktail | Roche Tablets |  |
| Pilocarpine | Cayman Chemicals |  |
| PMSF | Sigma Aldrich |  |
| Potassium chloride | Sigma Aldrich |  |
| Potassium dihydrogen phosphate (KH_2_PO_4_) | Sigma Aldrich |  |
| Propidium Iodide | Sigma Aldrich |  |
| Protein ladder | Abcam |  |
| Pyridine | SRL, India |  |
| Rapamycin | Cayman Chemicals |  |
| Rhodamine 123 | Sigma Aldrich |  |
| Rubbing alcohol (70%) | Sigma Aldrich |  |
| Skimm milk | Sigma Aldrich |  |
| Sodium chloride | Sigma Aldrich |  |
| Sodium deoxycholate | Sigma Aldrich |  |
| Sodium Dodecyl sulphate | Sigma Aldrich |  |
| Sodium Dodecyl Sulphate (SDS) | SDFCL, India |  |
| Sodium hydroxide | Sigma Aldrich |  |
| Sodium Valproate | Sigma-Aldrich |  |
| TBA (2-thiobarbituric acid) | SDFCL, India |  |
| TCA (Trichloroacetic acid) | SDFCL , India |  |
| Thiazoyl blue tetrazolium bromide (MTT) | Sigma-Aldrich |  |
| TMED | Sigma-Aldrich |  |
| Tris | Sigma Aldrich |  |
| Trypsin-EDTA | Sigma Aldrich |  |
| Tween-20 | Sigma Aldrich |  |
| β-mercaptoethanol | Sigma Aldrich |  |

###

### **Instruments and apparatus:**

| **Instruments/Apparatus** | **Company** |
| --- | --- |
| -20 degree Refrigerator | Elanpro |
| 4 degree refrigerator | Cellfrost |
| Autoclave | Tomy SX-500 |
| Bench-top Shaker | Tarsons |
| Biosafety Hood | Labcono logic A2 biosafety cabinet |
| Blot imaging system | UVP biosystem @515 imaging system |
| Cell imaging system | Biotek Cytation 5 imaging system |
| Centrifuge | Thermo scientific legend micro 21R |
| Deep Freezer | Sigma 3-30K, India |
| Dry bath | Sigma Aldrich |
| Electrophoresis Assembly | Bio Rad power pack basic |
| Elevated plus maze | Fabricated in our laboratory |
| ELISA reader | Electronics Corporation of India Ltd., India |
| Fluorescence Microscope | Nikon, Japan |
| Homogenizer | Remi, India |
| Ice flaking machine | Allied Frost |
| Incubator | New Brunswick galaxy, 48R, Eppendorf, India |
| Micro-spin | Spinnex |
| Micropipettes | Eppendorf, Germany |
| Micropipettes | Thermo fisher |
| Milli-Q assembly | Milli-Q®, Millipore |
| Morris water maze (Panlab Harvard apparatus with SMART v3.0.03 software) | Harvard, USA |
| pH meter | Meter Toledo |
| Rota Rod Apparatus | Harvard Panlab apparatus |
| Transfer apparatus | BioRad Transblot Turbo |
| Vacuum pump | DLR |
| Vortex mixer | Corning, Sigma Aldrich |
| Water bath | Thermo scientific |
| Weighing balance | Citizen Scale Pvt Ltd., India |

### **ELISA Kits:**

| **ELISA Kits** | **Company** | **Cat. No.** |
| --- | --- | --- |
| Mouse phospho-NF-κB ELISA kit | Cusabio Technology | CSB-EL5055 |
| Mouse IL-1β ELISA kit | Krishgen Biosystems, India | KB2063 |
| Mouse TNF-α ELISA kit | Krishgen Biosystems, India | KB2145 |
| Mouse IL-6 ELISA kit | Krishgen Biosystems, India | KB2068 |
| Mouse TGF-β1 ELISA kit | Cusabio Technology | CSB-E04726M |
| Mouse TGF-β2 ELISA kit | Cusabio Technology | CSB-EL023452MO |
| Mouse TGF-β3 ELISA kit | Cusabio Technology | CSB-EL023453MO |
| LDH diagnostic kit | Randox, UK | LD3842 |
| Mouse LDH assay kit | Abcam, USA | ab102526 |
| CaspGLOW^TM^ Fluorescein Active Caspase-3 Staining Kit | Biovision, USA | K183-25, -100 |
| In-Cell Phospho-AKT/AKT ELISA Colorimetric Detection Kit | Pierce, Thermo Scientific USA | 62215 |
| In-Cell Phospho-ERK/ERK ELISA Colorimetric Detection Kit | Pierce, Thermo Scientific USA | 62206 |
| Mouse Phospho-AKT (S473) and total AKT ELISA kit. | Ray biotech | PEL-AKT-S473-T-1 |
| Mouse phospho- p44/42 Erk1/2 (Thr202/Tyr204) and total Erk1/2 ELISA Kit | Ray biotech | 7050 |
| Mouse phospho-p70S6 kinase (Thr 389) ELISA kit | Cell signaling Technology | #7038 |
| Mouse total-p70S6 kinase ELISA kit | Cell signaling Technology | #7063 |

**4.1.4. Antibodies:**

| **Antibodies** | **Company** | **Catalogue number** |
| --- | --- | --- |
| Mouse p53 Antibody | Santa cruz biotechnology | sc-126 |
| Mouse beta actin antibody | Novus Biologicals | NB600-501 |
| Ranbit anti-mouse IgG H&L (HRP) | Abcam | ab6728 |

**Softwares:**

| **Software** | **Company** |
| --- | --- |
| G*Power Software (version 3.1.9.3 for Mac OS) | Axel Buchner, Universität Düsseldorf, Germany |
| GraphPad Prism (version 9.0 for Mac OS) | GraphPad software, USA |
| Endnote X9 for Macintosh^®^. | Clarivate Analytics, USA |
| Chemdraw Professional 16.0 | Perkin Elmer, USA |
| Image J (version 1.3) | National Institutes of Health, USA |
